# Easy *C. elegans* Worm Lifespan Plotting and Statistics with WLSplot

**DOI:** 10.17912/micropub.biology.000991

**Published:** 2023-10-02

**Authors:** Blaise L Mariner, Jack Peterson, Jackson C Taylor, Alex T Achusim, Mark A McCormick

**Affiliations:** 1 Biochemistry and Molecular Biology, University of New Mexico, Albuquerque, New Mexico, United States; 2 Chemical and Biomedical Engineering, University of New Mexico, Albuquerque, New Mexico, United States

## Abstract

WLSplot is an R package used to easily analyze lifespan survival data, and display results graphically as a survival curve with useful labels and statistical information auto-generated from the data and added to the graph, within a single function. It is designed primarily with *Caenorhabditis* *elegans* lifespan data in mind initially but can easily be used for other types of survival data. The WLSplot GitHub repository provides a blank template spreadsheet to be used for collecting lifespan data, instructions on how to install and run WLSplot, and examples covering RNAi, Genotype, or Drug lifespan experimental set-ups. WLSplot can analyze and plot multiple experiments in bulk while correctly italicizing worm gene names and adding asterisks and p-values to the plot legend when a significantly different lifespan from the designated control lifespan is seen. This is returned as an editable scalable vector graphics (svg) file for each output, and WLSplot can also return the summary of the directly plotted data so that the researcher can do their own further manipulation, in addition to being able to edit the output svg files.

**Figure 1. Example plot generated from WLSplot workflow:**
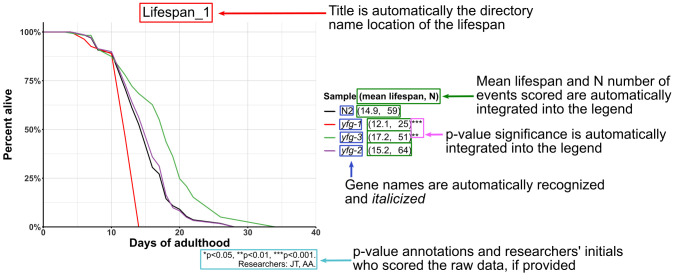
Visualization of WLS_autoplot() features when plotting worm lifespan data with the WLSplot R package.

## Description

Multiple genetic pathways have been found that can delay aging and extend lifespan of some aging model organisms by 10-fold, through pathways conserved to mammals (Ayyadevara et al., 2008; Tissenbaum & Guarente, 2002) . Interest in understanding aging using simple model organisms accelerated upon the finding of *C. elegans* mutants that drastically extend lifespan (Klass, 1983; Friedman & Johnson, 1988; C. Kenyon et al., 1993) . The foundation of the work done by researchers studying model organisms of aging such as the yeast *S. cerevisiae* and the worm *C. elegans* has brought the field to a deeper understanding of the aging process in humans, as exemplified by the genetic screens done to find aging pathways in both organisms (Ayyadevara et al., 2008; Gems & Partridge, 2013; C. J. Kenyon, 2010; McCormick & Promislow, 2017; Smith et al., 2008; Tissenbaum & Guarente, 2002) .

Research into model organisms has shown that many mutations can impact lifespan (Derisbourg et al., 2021; Lithgow & Walker, 2002; McCormick et al., 2015) . The aging field is moving rapidly to automate worm lifespan measurement with robotics in an attempt to more deeply define aging pathways (Felker et al., 2020) . Worms have already been shown to have conserved aging pathways to *Drosophila* and mammals (Guarente & Kenyon, 2000; Smith et al., 2008; Martin-Montalvo et al., 2013) . Data are traditionally collected through the manual prodding of organisms and each day the researcher records how many organisms are alive, dead, or censored (often worms that are missing, bagged or exploded are censored) on a data collection spreadsheet. Upon the completion of a lifespan, the data is then plotted using Kaplan-Meier survival curves (Kaplan & Meier, 1958) . Statistical significance is commonly calculated by using the Wilcoxon Rank-Sum test (Wilcoxon, 1946) .

The W orm L ife s pan plot (WLSplot) github package v1.0 (https://github.com/labmccormick/WLSplot) has the ability to automate the Kaplan-Meier survival curve plotting and Wilcoxon Rank-Sum statistic calculations from worm lifespans. WLSplot uses R (currently R v 4.3.1), a programming language with growing popularity in the life and data sciences (Dalgaard et al., 2010) . WLSplot’s main function, WLS_autoplot(), takes lifespan data from the datasheet (WLSplot provides a blank sample spreadsheet) containing the experimental data and saves the plot out with the experimental variables automatically integrated ( **Figure 1** ). The title is automatically the directory in which the lifespan datasheets exist. The mean lifespan, n, and associated p-values in comparison to the control are automatically integrated into the legend, while WLSplot attempts to automatically identify *C. elegans* gene names for automatic correct italicization. A caption that describes the p-values of the asterisk annotations on the plot. The caption can also credit the researchers who collected the lifespan data by initials and records the temperature of the lifespan. The user has the ability to easily change the colors of the conditions plotted as well. Lastly, WLS_autoplot() has the ability to batch process multiple daughter directories if the researchers have several lifespans that need to be analyzed at once.

WLS_autoplot() has the ability to write-out the plotted data used to generate the Kaplan-Meier survival curve svg, or just return the data without writing the data out to a flat file. WLS_autoplot() can also automatically output individual plots of each condition that shows significantly changed lifespan in comparison to the control, for easy visualization of these specific individual conditions, without changing the color of the line in comparison to the original lifespan plot. All plots are saved as editable svg vector graphics files, using the svglite R library. This file type has great advantages: a vector graphics editing program such as the free and open source Inkscape can be used to modify individual text and graphical elements of the plot by hand as needed ( *Inkscape Project* , 2020).

To get started, the new user must install R as well as the WLSplot R package and any of its R package dependencies that are not already installed, as outlined in detail on the main page of the github repository and in the linked video tutorials. Once installed, there are several examples provided that show how the user can analyze RNAi, mutant, drug, or other treatment styles of lifespan experiments. WLSplot also contains two other functions that may be useful for researchers in processing worm lifespan data. WLSplot can import data from comma-separated values (csv), OpenDocument spreadsheet (ods), or Excel (xlsx) files, and example files as well as blank lifespan scoring template files are provided for all of these formats. First, WLS_convert_counts() provides researchers the ability to easily convert and annotate basic lifespan spreadsheets to flattened files containing the data that then can be used to plot the desired Kaplan-Meier survival curves. Second, WLS_manualplot() gives the user the ability to pass in processed data for more specific control of details such as the plot title, x-axis limits, and line colors.

Previously published *C. elegans* lifespan software includes OASIS and OASIS 2, which offer survival-curve plotting ability allowing users to upload their spreadsheets with a user-friendly web interface (Han et al., 2016; Yang et al., 2011) . OASIS 2 also includes the option to calculate statistics inquiring into the differences in maximum lifespan between samples. SurvCurv is another previously published method of lifespan plotting, that has a user-friendly web interface that takes user-uploaded data and produces plots of these data (Ziehm et al., 2015) . SurvCurv is also a database, where users can upload, store, and share their lifespan analyses. WLSplot supplements these two tools, but breaks new ground by plotting survival curves from one or multiple experiments quickly, by-passing the need for the experimenter to handle and upload data files to a web browser, and implementing these features in provided open source R code that might allow easy additional customization by users so inclined.

WLSplot was written for biologists to automate their lifespan plotting and limit their need to work within a spreadsheet, allowing these researchers to increase their time at the bench. WLSplot is not limited to worm lifespan assays, and can be used for other physiological assays that result in survival curves, such as thermotolerance, oxidative stress assays, and survival on pathogens (Park et al., 2017) . WLSplot will aid researchers in quickly analyzing data that is to be plotted on a survival curve.

## Extended Data

Description: software Package WLSplotv1.0. Resource Type: Software. DOI: 10.22002/ke80f-8ac39
